# Maslinic acid protects against pressure-overload-induced cardiac hypertrophy by blocking METTL3-mediated m^6^A methylation

**DOI:** 10.18632/aging.203860

**Published:** 2022-03-28

**Authors:** Ming Fang, Jun Deng, Qiangping Zhou, Zhengbang Hu, Lixia Yang

**Affiliations:** 1 Department of Emergency, The First Affiliated Hospital of Nanchang University, Nanchang, Jiangxi, China; 2 Department of Infectious Disease, The First Affiliated Hospital of Nanchang University, Nanchang, Jiangxi, China

**Keywords:** myocardial hypertrophy, maslinic acid (MA), N6-methyladenosine (m^6^A), methyltransferase-like 3 (METTL3)

## Abstract

Coordinated response of the heart to physiological stressors (including stress overload, ischemia, hypothyroidism, and metabolic signals) is a hallmark of heart disease. However, effective treatment and its molecular targets are unknown. Although Maslinic Acid (MA) has been shown to inhibit inflammatory responses with strong anti-tumor, anti-bacterial, and antioxidant effects, information on its role and underlying mechanism in cardiac hypertrophy are scanty. The present study revealed that 10-10^3^ μg/ml MA treatment significantly inhibited Ang-II induced hypertrophy in NMCMs and the dosage did not influence the cell viability of H9C2 and NCMCs. Moreover, the anti-hypertrophy effect of MA (30 mg/kg·day) was verified in the TAC-induced hypertrophy mouse model *in vivo*. Further analysis showed that MA administration decreased the total RNA m^6^A methylation and METTL3 levels in Ang-II treated NMCMs and TAC stressed hearts. Rescue experiments under adenovirus-mediated myocardial METTL3 overexpression confirmed that METTL3-mediated m^6^A methylation is essential in M-driven inhibition of myocardial hypertrophy. Collectively, MA exerts a significant anti-hypertrophy effect by regulating the modification of METTL3-mediated m^6^A methylation *in vitro* and *in vivo*. These findings may provide a platform for establishing a new target and strategy for cardiac hypertrophy treatment.

## INTRODUCTION

Cardiac hypertrophy is a common physiological compensatory response of the heart to multiple stressors aimed at maintaining normal cardiac function. Cardiac enlargement due to myocardial injury, hypertensive stress, or excessive neurohumoral activation is classified as pathological hypertrophy and has been demonstrated to be associated with poor adaptive remodeling and cardiac dysfunction [1]. Pathological cardiac hypertrophy is a major risk factor for cardiomyopathy, heart failure, and sudden cardiac death [2]. The understanding of pathological regulatory factors of cardiac hypertrophy has been improved; however, the molecular mechanism of cardiac hypertrophy remains unclear. Therefore, more effective treatment methods and intervention targets are urgently needed.

Maslinic acid (MA) is a pentacyclic triterpenoid rich in olive pericarp with a wide range of pharmacological properties [3]. The potential of MA has been demonstrated in blood glucose reduction, inhibition of oxidative damage, and induction of apoptosis of human colon cancer cells. Mounting evidence indicates that MA exerts anti-inflammatory and anti-arthritis effects by inactivating the nuclear factor Kappa-B (NF-κB) [4–10]. Also, MA decreases the expression of hypoxia-inducible factor-1 in lung cancer cells and protects against many cardiovascular diseases [11, 12]. Recent evidence shows that MA can prevent hypertrophy and fibrosis caused by pressure overload and improve cardiac function in mice undergoing aortic banding surgery [12]. Studies have also demonstrated that MA potentially reduces phosphorylation of protein kinase B and extracellular regulatory protein kinase in hypertrophic hearts, decreases cardiac hypertrophy, and inhibits the activation of AKT and ERK signaling pathways *in vitro* [12]. However, the underlying mechanism of MA on pressure-overload-induced cardiac hypertrophy and its specific regulatory mechanism is largely elusive.

N6-methyladenosine (m^6^A) is the most common post-transcriptional modification of mRNA in mammals [13]. Recently, numerous studies have revealed a critical role for m^6^A in the regulation of various biological processes, including embryo development, cell differentiation, regeneration, and tumorigenesis [14–26]. However, a few studies are related to m^6^A in the cardiovascular field. Several pieces of evidence show that the overall level of m^6^A is elevated in myocardial infarction and ischemia-reperfusion injury, whereas the decrease of m^6^A potentially promotes autophagy flow and improves cardiac function [27–29]. However, the underlying mechanism by which m^6^A modifications impact cardiac function remains unclear.

Therefore, in the present study, based on *in vitro* and *in vivo* experiments, we purpose to explore the role and mechanism of m6A in cardiac hypertrophy inhibition from the perspective of m^6^A methylation modification.

## RESULTS

### MA attenuates the Ang-II induced NMCMs hypertrophy *in vitro*

**Figure 1:**
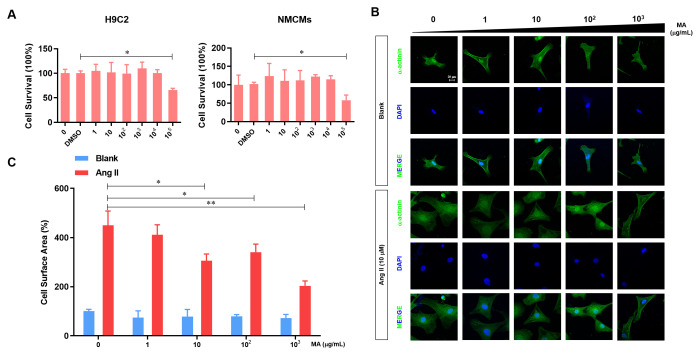
**MA-mediated inhibition of the Ang-II induced NMCMs hypertrophy *in vitro*.** (**A**) CCK-8 assay detection of the effect to MA on the cell viability of H9C2 and NMCMs; (**B**) Analysis of the effect of MA on the cardiomyocyte hypertrophy morphology after immune fluorescence staining with α-actinin and DAPI; (**C**) Quantitative analysis of cardiomyocyte surface area by image J software; *P<0.05, **P<0.01 compared to the indicated group.

H9C2 cells and NMCMs were sued to evaluate the cytotoxicity of MA *in vitro*. Our analysis demonstrated no influence on the viability of H9C2 cells and NMCMs at a dose of MA less than 10^4^ μg/ml, but cell viability decreased significantly when we increased the MA dosage raised to 10^5^ μg/ml (Figure 1A). To test the potential impact of MA on NMCMs hypertrophy, MA (0, 1 10^2^, and 10^3^ μg/ml) were added to the culture medium of NCMCs with or without the Ang-II treatment. Results revealed no influence of MA treatment on the cell morphology of NCMCs (Figure 1B). Ang-II induced the enlargement of NCMCs, suggesting a hypertrophy morphology. On the other hand, treatment with MA at 10-10^3^ μg/ml dosage dramatically inhibited Ang-II- induced hypertrophy of NMCMs. Similar results were found through quantitative analysis of the cell surface area in which 10-10^3^ μg/ml MA treatment significantly inhibited the Ang-II induced hypertrophy of NMCMs (Figure 1C).

### MA protects against TAC-induced cardiac hypertrophy *in vivo*

**Figure 2:**
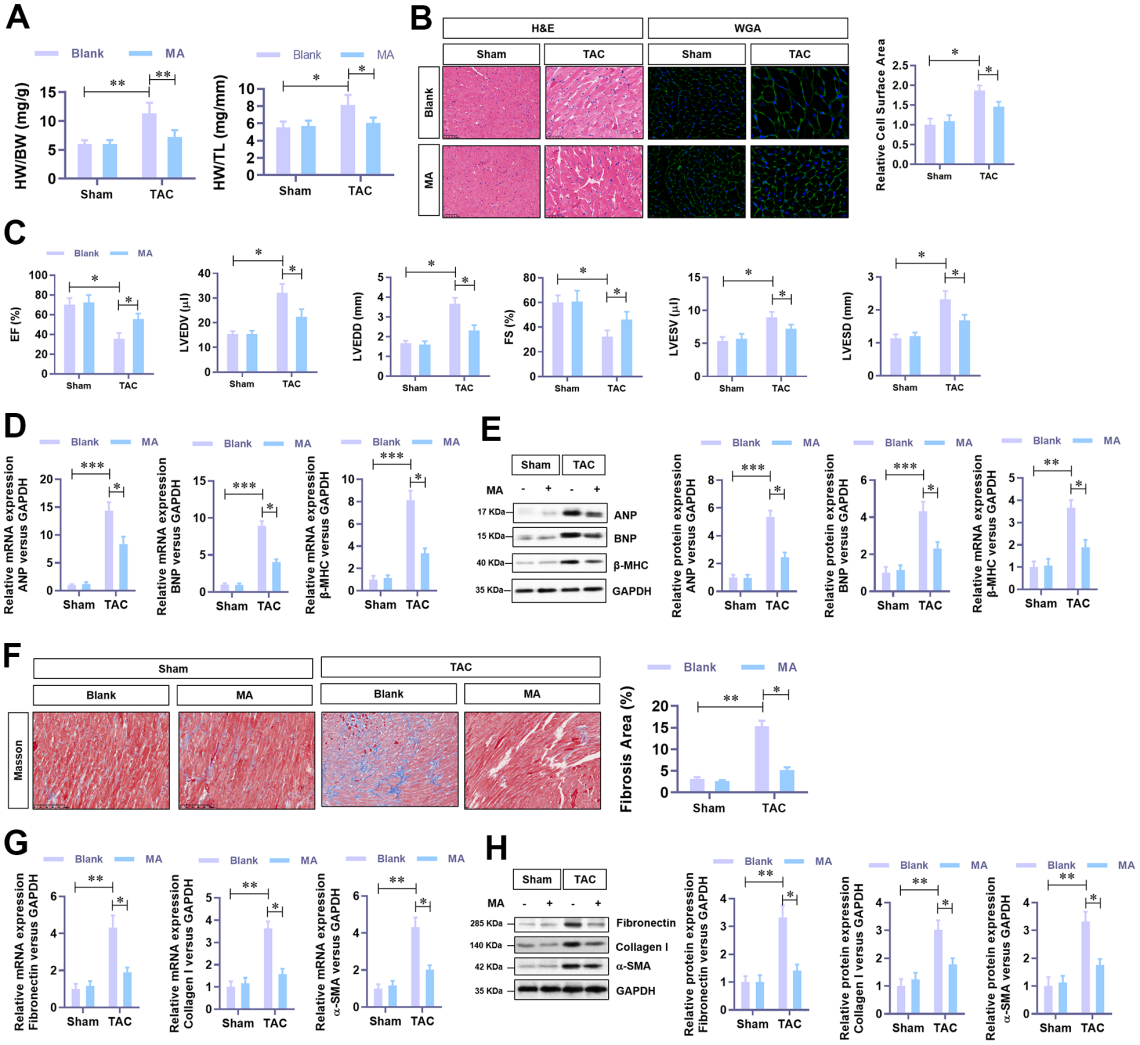
**The protective role of MA against TAC-induced cardiac hypertrophy *in vivo*.** (**A**) The ratio of HW/BW and HW/ TL, showing the effect of MA on the morphology of cardiac morphology. (**B**) Analysis of the effect of MA on the cardiomyocyte area in left ventricle through Histology H&E and WGA staining of cardiac cross-sections. (**C**) Echocardiography detection of the parameters of LVEF, LVFS, LVEDV, LVESV, LVEDD, and LVESD to evaluate the effect of MA on the cardiac function of TAC mice. (**D**) Real-time PCR analysis of mRNA levels of the hypertrophy markers (ANP, BNP, and β-MHC). (**E**) Western blot analysis of the protein levels of the hypertrophy markers (ANP, BNP, and β-MHC). (**F**) Masson staining detection of the effect of MA on TAC-induced myocardial fibrosis. (**G**) Real-time PCR analysis of mRNA levels of the myocardial fibrosis (Fibronectin, Collagen I, and α-SMA). (**H**) Western blot analysis of the protein levels of the myocardial fibrosis (Fibronectin, Collagen I and α-SMA); *P<0.05, **P<0.01 compared to the indicated group.

In this experiment, MA (30 mg/kg·day) was administrated in TAC mice to evaluate the effect of MA in TAC-induced cardiac hypertrophy. Results demonstrated that TAC elevated HW (heart weight)/BW (bodyweight) and HW (heart weight)/Tibal Length (TL) decreased significantly following MA treatment. Histological analysis (H&E and WGA staining) of cardiac cross-sections revealed a noticeable decrease in the cardiomyocyte area in the left ventricle in the MA treated TAC mice (Figure 2B). Cardiac function was assessed via Echocardiography using the parameters, LVEF, LVFS, LVEDV, LVESV, LVEDD, and LVESD were detected. Notably, MA treatment improved dramatically the parameters of TAC-impaired LVEF and LVFS but reduced the parameters of TAC elevated LVEDV, LVESV, LVEDD, and LVESD; these results suggested an improved cardiac function in MA treated TAC mice (Figure 2C). Moreover, the TAC elevated mRNA (Figure 2D) and protein (Figure 2E) levels of the hypertrophy markers in LV tissues, including ANP, BNP and β-MHC decreased significantly following MA treatment. Accordingly, Masson staining results demonstrated that MA treatment inhibited the TAC-induced myocardial fibrosis (Figure 2F), which was confirmed by decreased mRNA (Figure 2G) and protein (Figure 2H) expression of Fibronectin, Collagen I, and α-SMA. These results demonstrate an anti-hypertrophy role of MA *in vivo*.

### MA significantly impairs the elevated level of m^6^A methylation and METTL3 in hypertrophic cardiomyocytes *in vitro* and *in vivo*

**Figure 3:**
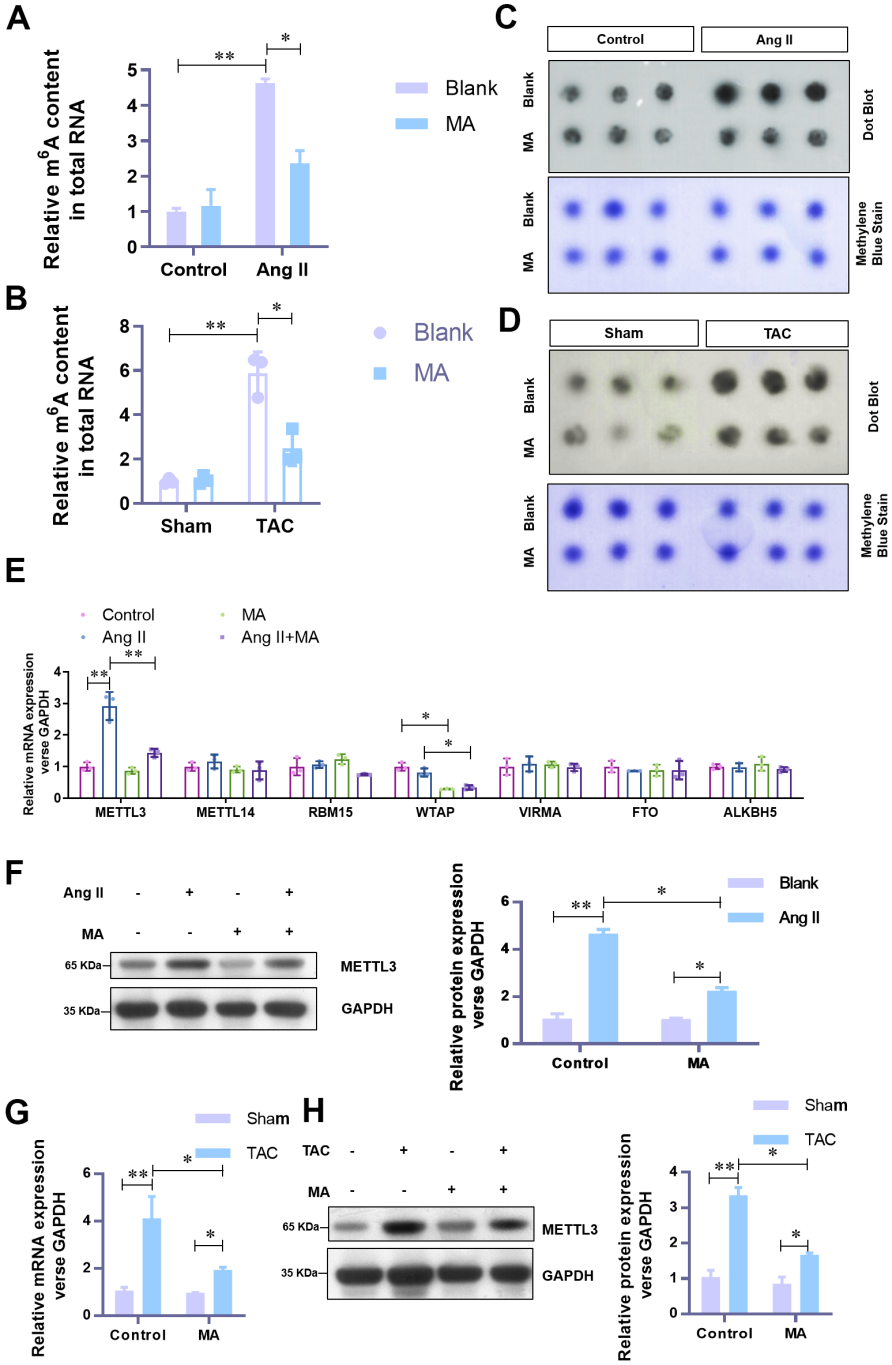
**MA-induced significant impairment of the elevated level of m^6^A methylation and METTL3 in hypertrophic cardiomyocytes *in vitro* and *in vivo*.** (**A**) The effect of MA on the total RNA m^6^A methylation content in Ang-II induced hypertrophic NMCMs and (**B**) TAC induced hypertrophic LV tissues (detected with the EpiQuik m^6^A RNA Methylation assay kit). (**C**) The effect of MA on the total RNA m^6^A methylation content in Ang-II induced hypertrophic NMCMs and (**D**) TAC induced hypertrophic LV tissues (detected with by Dot Blot). Methylene blue staining served as a loading control. (**E**) Real-time PCR analysis of the mRNA expression levels of the major methyltransferases (METTL3, METTL14, RBM15, WTAP, VIRMA) and demethylases (FTO and ALKBH5). (**F**) Western blot analysis of the effect of MA on the protein level of METTL3 in Ang-II induced hypertrophic NMCMs. (**G**) Real-time PCR analysis of the effect of MA on the protein level of METTL3 in TAC induced hypertrophic LV tissues. (**H**) Western blot detection of the effect of MA on the protein level of METTL3 in TAC-induced hypertrophic LV tissues; *P<0.05, **P<0.01 compared with the indicated group.

To understand the underlying mechanism of the anti-hypertrophy role of MA, attempts were made to explore the effect of MA on m^6^A methylation. Previous evidence shows that the elevated level of m^6^A methylation is critical to the hypertrophic progress of cardiomyocytes. In our analysis, with the MA treatment, elevated total RNA m^6^A methylation content in Ang-II induced hypertrophic NMCMs (Figure 3A) and TAC induced hypertrophic LV tissues (Figure 3B) decreased significantly. These results were further confirmed by Dot Blot (Figure 3C, 3D). To identify the critical contributing factor to the impaired m^6^A methylation, mRNA expression levels of the major methyltransferase or demethylase, including METTL3, METTL14, RBM15, WTAP, VIRMA, FTO, and ALKBH5 were assessed. Results showed a dramatic decrease in the mRNA level of METTL3 when Ang-II induced hypertrophic NMCMs were treated with MA (Figure 3E). The decrease of protein level of METTL3 was validated by Western blotting (Figure 3F). Besides, decreased mRNA and protein levels of METTL3 in MA treated TAC mice were verified by real-time PCR (Figure 3G) and Western blotting (Figure 3H). These findings suggest a potential critical contribution of METTL3 mediated m^6^A methylation to the anti-hypertrophy effect of MA.

### METTL3 over-expression reverse the anti-hypertrophy effect of MA *in vivo*

**Figure 4:**
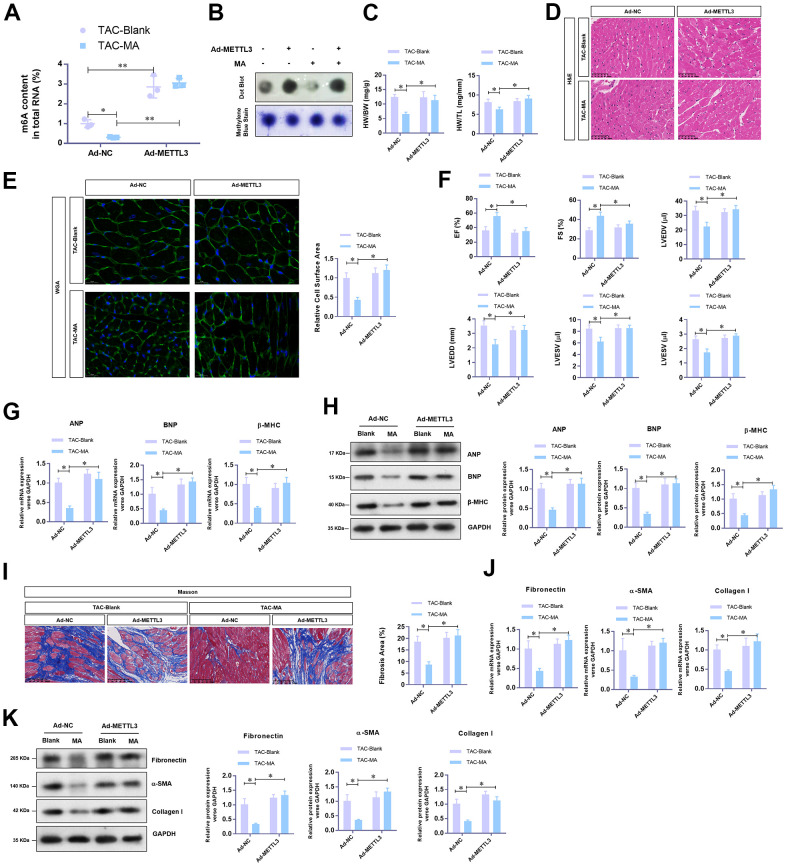
**The reverse effects of METTL3 over-expression on the anti-hypertrophy effect of MA *in vivo*.** (**A**) The effect of METTL3 over-expression on the MA impaired total RNA m^6^A methylation content in TAC induced hypertrophic LV tissues detected with the EpiQuik m^6^A RNA Methylation assay kit and (**B**) confirmed by Dot Blot. (**C**) The ratio of HW/BW and HW/ TL shows the effect of METTL3 over-expression on the MA inhibited morphology of cardiac morphology. (**D**) Analysis of the effect of METTL3 over-expression on the MA preserved cardiomyocyte area in left ventricle through Histology H&E and (**E**) WGA staining of cardiac cross-sections. (**F**) Echocardiography detection of the parameters of LVEF, LVFS, LVEDV, LVESV, LVEDD, and LVESD to evaluate the effect of METTL3 over-expression on the MA preserved cardiac function of TAC mice. (**G**) Real-time PCR analysis of the mRNA levels of the hypertrophy markers (ANP, BNP, and β-MHC). (**H**) Western blot detection of the protein levels of the hypertrophy markers (ANP, BNP, and β-MHC). (**I**) Detection of the effect of MA on the TAC-induced myocardial fibrosis via Masson staining. (**J**) Real-time PCR analysis of the mRNA levels of the myocardial fibrosis (Fibronectin, Collagen I, and α-SMA). (**K**) Western blot analysis of the protein levels of the myocardial fibrosis (Fibronectin, Collagen I and α-SMA); *P<0.05, ** *P* <0.01 compared to the indicated group.

To unravel the critical role of METTL3 mediated m^6^A methylation in the anti-hypertrophy effect of MA, adenovirus-mediated over-expression of METTL3 was achieved in TAC mice with or without MA treatment. Total RNA m^6^A methylation content was appreciably increased by METTL3 in LV tissues from TAC mice despite treatment with MA (Figure 4A); these results were verified by Dot Blot (Figure 4B). As expected, METTL3 over-expression significantly reversed the MA decreased HW/BW and HW/TL (Figure 4C). Consistent findings were reported by histology H&E (Figure 4D) and WGA staining of cardiac cross-sections, whereby the MA decreased cardiomyocyte area in the left ventricle was enlarged by METTL3 over-expression in TAC mice (Figure 4E). Regarding cardiac function, METTL3 over-expression reduced the LVEF and LVFS to a similar level with the control TAC group in MA-treated mice (Figure 4F). Furthermore, METTL3 over-expression increased the parameters of LVEDV, LVESV, LVEDD, and LVESD, suggesting an impaired cardiac function (Figure 4F). Similar findings were reported that METTL3 over-expression increased mRNA (Figure 4G) and protein (Figure 4H) levels of the hypertrophic markers in MA treated TAC mice. Also, the Masson staining demonstrated induction of myocardial fibrosis by METTL3 over-expression in MA treated TAC mice (Figure 4I), which was evident by the elevated mRNA (Figure 4J) and protein (Figure 4K) expression of Fibronectin, Collagen I, and α-SMA. Taken together, these results demonstrate a protective role of MA against pressure-overload-induced cardiac hypertrophy by blocking METTL3-mediated m^6^A methylation.

## DISCUSSION

Several researchers have demonstrated the potential of MA in blood glucose reduction, inhibition of oxidative damage, and induction of apoptosis of human cancer cells [4–8]. However, information about the role and underlying mechanism of MA in cardiac hypertrophy are scarce. The present investigation found that 10-10^3^ μg/ml MA treatment significantly inhibited the Ang-II induced hypertrophy in NMCMs, in which dosage the cell viability of H9C2 and NCMCs were not affected. Further investigation validated the anti-hypertrophy effect of MA (30 mg/kg·day) in the TAC-induced hypertrophy mouse model *in vivo*, evidenced by the reduced HW/BW, HW/TL, cardiomyocyte area, hypertrophic markers expression, and preserved cardiac function.

The RNA m^6^A modification is part of the larger field of RNA epigenetics, an evolving field that has only just begun to be explored in the heart [27–29]. Like the deposition of epigenetic markers on DNA, mRNA methylation potentially influences gene expression and determines the fate of mRNA subsets [21]. This is particularly crucial for stress response pathways requiring rapid response to environmental stress [19].

The present investigation revealed that Ang-II and TAC elevate the total RNA m^6^A methylation and METTL3 levels in isolated NMCMs and hearts respectively. These results are consistent with previous findings. Intriguingly our analysis found that MA administration appreciably decreased the total RNA m^6^A methylation and METTL3 levels in Ang-II treated NMCMs and TAC stressed hearts. Moreover, through adenovirus-mediated myocardial METTL3 overexpression, the present study demonstrated that METTL3-mediated m^6^A contributes to MA-driven inhibition of myocardial hypertrophy.

In conclusion, MA exerts a significant anti-hypertrophy effect, via a mechanism related to the regulation of METTL3-mediated m^6^A methylation modification. These findings may provide clues in establishing a new target and strategy for cardiac hypertrophy treatment.

## MATERIALS AND METHODS

### Animals

Male C57BL/6 (8-week-old, 20-25 g) was purchased from SLAC Laboratory Animal Center (Shanghai, China) and reared under a sterile environment in the animal facility of the First Affiliated Hospital of Nanchang University. All mice were fed at room temperature (22±2° C), humidity at 60%, and light-dark cycle for 12 h for at least 1 week before the experiment. All animal experimental procedures followed the Guidelines of the Animal Care and Use Committee. The research on animals adhered to relevant national laws and regulations on animal care and use. The study was approved by the Animal Ethics Committee of the First Affiliated Hospital of Nanchang University.

### Animal model

A transverse aortic constriction (TAC) model was established to mimic pressure-overload-induced cardiac hypertrophy [27]. Briefly, mice were anesthetized with isoflurane and subjected to open-heart surgery. The aorta was dissected and tied with a 0-7 silk thread around the vessel and a 26-gauge needle to ensure consistent occlusion. Thoracotomy and aortic dissection without aortic coarctation were administered to mice in the sham operation group. To overexpress the level of METTL3, METTL3 overexpression adenoviruses and its negative controls were purchased from Genelily (Shanghai, China) and injected into mice in the heart 1 week before the TAC operation (5×10^7^ viral particles). Mice in the Maslinic acid (MA) treatment group were intraperitoneally injected with MA (30 mg/kg daily) for 2 weeks, with the same volume of normal saline as control. Echocardiography was performed with a Visualsonics Vevo 2100 ultrasound system (Toronto, Canada) after 4 weeks of surgery. Parameters, including LVEF (Left Ventricular Ejection Fractions), LVFS (Left Ventricular Fractional shortening), LVEDV (Left Ventricular End Diastolic Volume), LVESV (Left Ventricular End Systolic Volume), LVEDD (Left Ventricular End Diastolic Dimension), and LVESD (Left Ventricular End Systolic Dimension) were analyzed. Eventually, the hearts were collected and further analyzed.

### Histology

The isolated hearts from mice were fixed with 10% formalin, dehydrated, and embedded in paraffin. Five histopathology sections were acquired and stained with hematoxylin-eosin (H&E) and wheat germ agglutinin (WGA, Alexa Fluor 488 conjugated). The cross-sectional area of cardiomyocytes was determined via Thermo Fisher staining. Collagen deposition was evaluated by Masson (HT15-1KT, Sigma Aldrich) staining kit following the manufacturer's protocol. Section microscopy was performed. The fibrosis area based on myocardial collagen area/visual field area was determined using the Image Pro-Plus 6.0 Image analysis software.

### Cell culture

The heart from the neonatal mouse was divided into small pieces and the resultant tissue was digested in HEPES buffer saline at 37° C. Next, 10% FBS was used to neutralize the trypsin. Cells were centrifuged(1000 rpm, 5 minutes) and suspended in DMEM/F12 (Invitrogen, Carlsbad, CA). To extract neonatal mouse cardiomyocytes (NMCM), the cells were inoculated on collagen-coated silica gel sheets for 24 h followed by 24 h culture in a serum-free medium. Rat cardiomyocytes H9C2 were purchased from the Chinese Academy of Sciences (Shanghai, China). *In vitro*, cardiac hypertrophy models were acquired from NMCMs cells treated with 1 mmol/L angiotensin II (Ang-II, Sigma-Aldrich, St. Louis, Mo) for 48 h.

### Cell viability assay

The viability of neonatal cardiomyocytes was detected by CCK-8 assay (Dojindo). Briefly, primary neonatal cardiomyocytes were inoculated in 96-well plates (10^3^ cells/well) and cultured in 100 μL DMEM/F12 medium for 24 h. Subsequently, cells were treated with different concentrations of MA (1, 10, 10^2^, 10^3^, 10^4^, and 10^5^ μM). Cell viability was measured using the CCK-8 kit following the manufacturer's instructions. Absorbance was measured by a microplate reader (Thermo, USA) at 450nm. All the experiments were performed in triplicate independently.

### Cell surface measurements

Neonatal cardiomyocytes were fixed, infiltrated, and stained with FITC labeled anti-α-actin antibody (Abcam, Cambridge, UK). Cells were incubated at 4° C overnight and then treated with DAPI staining for 10 minutes. Immunofluorescence images were recorded by a Fluorescence Microscope (Zeiss LSM 800). The surface area of neonatal cardiomyocytes was measured by the Image-Pro Plus 6.0 software.

### Real-time PCR

Total RNA was extracted from cells and heart tissue using Trizol reagent (Thermo Fisher). Subsequently, cDNA was synthesized by the Prime Script Reverse Transcription Reagent Kit (TOBYBO, Japan). The real-time PCR was performed in ABI Viia 7 real-time PCR system with the following primer pairs: ANP forward primer: 5’-CCCTGGGACCCCTCCGATAG-3’, ANP reverse primer: 5’-CGTGACACACCACAAGGGCT-3’; BNP forward primer: 5’-GCAGAAGCTGCTGGAGCTGA-3’, BNP reverse primer: 5’-TCCTGCAGCCAGGAGGTCTT-3’; β-MHC forward primer: 5’-GGCCAAGATCGTGTCCCGAG-3’, β-MHC reverse primer: 5’-ACTTGGGTGGGTTCTGCTGC-3’; Fibronectin forward primer: 5’-AGCTTCTCCAAGCATCGCCC-3’, Fibronectin reverse primer: 5’-GACACACAGCCACAGGCCAT-3’; Collagen I forward primer: 5’- CTGGTGCTCGCGGTAACGAT-3’, Collagen I reverse primer: 5’- CAGCACCAGGGTTTCCAGCA-3’; α-SMA forward primer: 5’-CCGGCTTCGCTGGTGATGAT-3’, α-SMA reverse primer: 5’-GTCGGATGCTCTTCAGGGGC-3’; GAPDH forward primer: 5’-TGTGGATGGCCCCTCTGGAA-3’, GAPDH reverse primer: 5’-TGACCTTGCCCACAGCCTTG-3’. The relative gene expression normalized to GAPDH gene expression was analyzed using the 2^−ΔΔCt^ method.

### Western blotting

Proteins are isolated from frozen tissues and cells. The proteins were denatured, separated in 12% SDS-PAGE, and transferred to the PVDF membrane. The membrane was blocked and incubated with primary antibodies (including anti-ANP, anti-BNP, anti-b-MHC, anti-Fibronectin, anti-Collagen I, anti-α-SMA, anti-METTL3, and anti-GAPDH) and HRP labeled Goat anti Rabbit secondary antibody. All the antibodies were purchased from Abcam (Abcam). Target proteins were visualized via enhanced chemiluminescence techniques (Tannon, Shanghai, China) and quantified by Quantity One software (Bio-Rad).

### Total m^6^A content analysis

The EpiQuik m^6^A RNA Methylation assay kit (P-9005-48, Epigentek) was employed to evaluate the content of total RNA m^6^A. Changes in total RNA m^6^A level were assessed by Dot Blot as previously described [17].

### Statistical analysis

Data were expressed as Mean ± SEM. 2-tailed t-test (unpaired) was applied for comparisons between two groups, whereas ANOVA followed by the post hoc Bonferroni test was for used multiple comparisons. All analyses were performed with GraphPad Prism® version 6.0 software (GraphPad Software, Inc., La Jolla, CA, USA).
